# A simple and effective approach to quantitatively characterize structural complexity

**DOI:** 10.1038/s41598-020-79334-7

**Published:** 2021-01-14

**Authors:** Gongqiao Zhang, Gangying Hui, Aiming Yang, Zhonghua Zhao

**Affiliations:** Research Institute of Forestry, Chinese Academy of Forestry, Key Laboratory of Tree Breeding and Cultivation of National Forestry and Grassland Administration, Beijing, 100091 China

**Keywords:** Forestry, Forest ecology

## Abstract

This study brings insight into interpreting forest structural diversity and explore the classification of individuals according to the distribution of the neighbours in natural forests. Natural forest communities with different latitudes and distribution patterns in China were used. Each tree and its nearest neighbours form a structural unit. Random structural units (or random trees) in natural forests were divided into different sub-types based on the uniform angle index (W). The proportions of different random structural units were analysed. (1) There are only two types of random structural units: type R1 looks similar to a dumbbell, and type R2 looks similar to a torch. These two random structural units coexist in natural forests simultaneously. (2) The proportion of type R1 is far less than that of R2, is only approximately 1/3 of all random structural units or random trees; R2 accounts for approximately 2/3. Furthermore, the proportion of basal area presents the same trend for both random structural units and random trees. R2 has approximately twice the basal area of R1. Random trees (structural units) occupy the largest part of natural forest communities in terms of quantity and basal area. Meanwhile, type R2 is the largest part of random trees (structural units). This study finds that the spatial formation mechanism of natural forest communities which is of great significance to the cultivation of planted forests.

## Introduction

One of the most influential tenets of forest management today is that natural forests are generally more structurally diverse than comparable younger stands or planted forests which leads to more reliable forest ecosystems^1^. The creation of stands with structural diversity has become the pivotal management objective in several countries^2,3^. Thus, this study aims to show more implications for forest managers interested in increasing the structural diversity of plantations.

Spatial structural diversity can be understood as an expression of environmental heterogeneity, biotic processes, or interactions between biotic and abiotic processes^4^. Structural diversity, or ‘structural heterogeneity’, ‘structural complexity’^5,6^ usually refer to the “naturalness”^6^, is one of the most indicative features of old-growth stands^5,7,8^; these features are significantly related to microclimatic variables and explicitly depends on spatial relations among neighbours^1^, which may influence the species diversity and regeneration in natural forests^9^. Studying spatial structural diversity is as important as animal/plant species diversity in forest communities^9^. To date, several studies have investigated the relationship between stand structural diversity and biodiversity^10–13^ or productivity^14,15^. Differences in stand management lead to differences in stand structural complexity^16^. More and more researchers and forest managers recognized now that increasing the complexity and diversity of forest stand structure is a possible way to support ecosystem sustainability, adaptability and resilience as well as biodiversity and productivity^6,17–19^.

Previous studies on environmental heterogeneity or structure diversity have mainly focused on the stand level, while few researchers have paid attention to the micro-characteristics of individuals. However, environments are not homogeneous, even at very fine scales^20–22^. Individuals and the spatial structure of neighbours are the basis of habitat heterogeneity and forest structure diversity, especially when studying ecological processes, such as tree recruitment, competition and death. These studies are most accurate when they are based on the analysis of individuals^23,24^. Studying the neighbourhood relationships of each tree can fully describe the intraspecific and interspecific interactions among plants. Pommerening and Uria-Diez^25^ and Wang et al.^26^ explored the effects of individuals with different diameters on plant diversity in neighbouring areas, it is believed that species differences affect the spatial distribution of populations. Other studies found that the individual density of neighbouring plants, individual density of the same species, relative plant size and relative species richness can significantly affect the growth of target species^27,28^. Recently, there have also been reports on the diversity of forest distribution patterns and their responses^26,29^. Several indices quantify spatial structure simultaneously by taking tree neighbours into account and intend to describe structural complexity at the α level^30–32^. These studies fully illustrate the importance of neighbours in describing spatial structure diversity.

Stand structural diversity is “essentially a measure of the number of different structural attributes present and the relative abundance of each of these attributes”^7^. Measuring structural complexity must be based on a quantitative description of the spatial structure of forest ecosystems, which has been challenging^32,33^. Most spatial methods take relative tree positions into account when describing the point pattern or quantitatively analysing stand spatial structure^34^. The positioning index of Clark and Evans is a method based on distance^35^. The Ripley's K-function, pair correlation function or O-ring function^36,37^, Voronoi polygon analysis^38^ or the uniform angle index method can also be applied for analysing forest patterns^39,40^. The uniform angle index method, which is based on the spatial relationship of the four nearest neighbouring trees, has unique advantages in guiding the spatial structure adjustment of forests and in simulating and reconstructing forest structure because it can be used to describe microstructure through both mean values and the frequency distribution^40–43^, which has been widely applied in stand structure analysis^44–46^.

Studies on the spatial structure diversity based on the uniform angle index have lasted for several years. Zhang et al.^47^ found that the proportions of trees with different neighbour distributions in natural forests: random trees (or random structural units) were the majority in both quantity and basal area, accounting for more than 50%. Clustered trees (or units) and even trees (or units) only accounted for a small proportion. However, plantations have no such characteristics with regular planting patterns. Almost all individual trees are uniformly distributed with their neighbours, most structures are even with low spatial structure diversity compared with natural forests. These ideas are inherent in planting policies^48^ in several countries, which may make the execution of planting activities, mechanized operations and harvesting more easier. Thus, our purpose of the series of studies is to find out the characteristics of the spatial structure diversity of natural forests and apply them to planted forests to improve the structural complexity and resilience of plantations.

### Objective

Currently, the most appropriate measure to describe the stand structural complexity is unknown, because different approaches may address different aspects of complexity. Setting up a framework and more sophisticated concepts of forest spatial distributions, variation and diversity are needed to develop the modern forest management^16,49^. Correctly describing, defining and classifying the spatial composition and diversity of natural forests can provide more clues for natural forest management and are of great significance for the near-natural transformation of planted forests. Therefore, taking natural forests in different regions of China as an example, this study analysed the composition characteristics of random structural units in natural forest communities to identify and quantify sub-categories within this class of structural units. The paper introduces and applies a novel way to characterize structural diversity and offer practical support to natural forest management and the near-natural cultivation of planted forests.

## Materials and methods

### Research areas

We analysed observations from 11 sample plots established in natural forests at different latitudinal zones in China. All live trees with a DBH (diameter at breast height) > 5 cm were tagged, and their positions were mapped with a Topcon GTS602 (Topcon Corporation, Tokyo, Japan) autofocus total station. The tree DBH, height, and crown diameter were measured. Table 1 provides general information on the plots^47^.

**Table 1:** Information on study plots.

Plot	Dimensions	Density trees/hm^2^	Number of species	Mean DBH/cm	Standard deviation of DBH	Basal area m^2^/hm^2^
A1	100 m × 100 m	924	1	20.0	7.27	32.90
A2	100 m × 100 m	1149	1	19.8	6.95	39.76
B3	200 m × 200 m	202	3	49.4	26.02	49.27
C4	100 m × 100 m	936	19	16.4	11.04	28.74
C5	100 m × 100 m	748	22	17.7	12.80	27.95
C6	100 m × 100 m	816	22	17.7	12.14	29.56
C7	100 m × 100 m	808	19	16.6	12.79	28.08
C8	100 m × 100 m	797	19	18.3	13.03	31.67
C9	100 m × 100 m	1178	20	14.7	10.66	30.73
D10	140 m × 70 m	888	49	16.1	11.03	26.53
E11	100 m × 30 m	820	85	23.5	17.34	54.87

**Figure 1:**
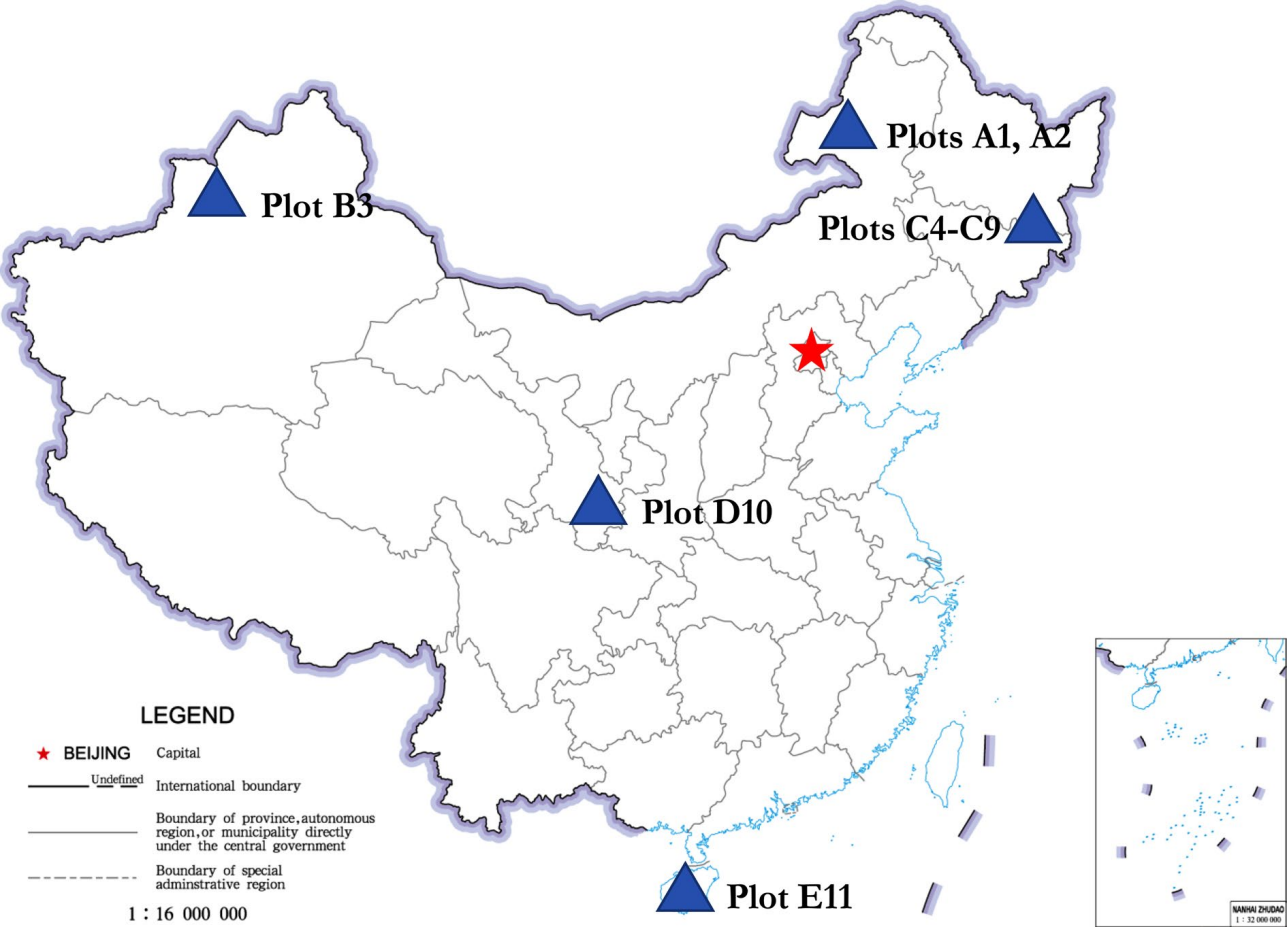
Location of the sample plots in China (The Chinese Map was generated by the Standard map online service, URL link: http://bzdt.ch.mnr.gov.cn).

These natural forests were distributed throughout five different latitudinal zones in China (Fig. 1). The forest types from north to south were as follows: *Pinus sylvestris* var. *mongolica*, two natural forests in sandy loam in a mid-temperate region (plots A1, A2); *Picea schrenkiana* var. *tianshanica*, a coniferous forest in a warm temperate region (plot B3); six *Pinus koraiensis*, broadleaf-conifer mixed forests in a warm temperate region (plots C4–C9); pine and oak mixed forest in a warm temperate and north subtropical transitional region (plot D10); and tropical montane rainforest (plot E11). None of the study forests had experienced any human disturbance^47^.

Plots A1 and A2, located in the southern part of a sandy area in Honghuaerji within the Inner Mongolian Province, belong to the transitional zone between the eastern slope of the middle of the Daxingan Mountains and the Inner Mongolian plateau (47° 36′–48° 35′ N, 118° 58′–120° 32′ E); the location is 700–1100 m above sea level. The zone experiences a mid-temperate, semi-humid, and semiarid continental monsoon climate, with a mean annual temperature of 1.5 °C and average annual precipitation of 344 mm. The main soil type is sand. The forest type is natural pure *Pinus sylvestris* var. *mongolica* forest^47^.

Plot B3, located in the Xitian Mountain National Nature Reserve in Gongliu County, Xinjiang Province, is part of the Tianshan Mountains (43° 59′–43° 28′ N, 87° 12′–87° 50′ E) and located 1635 to 1706 m above sea level. The region experiences a temperate continental climate, with mostly cold weather and great seasonal changes in temperature. The mean annual temperature is approximately 5 °C to 7 °C, and the average annual precipitation is 600 to 800 mm. The main soil type is mountain grey cinnamon forest soil, and the forest type is *Picea schrenkiana* natural forest with very few *Betula tianschanica*^47^.

Plots C4–C9, located on an eastern slope of the Jiaohe Forest Experimental Zone Management Bureau in Jilin Province (43° 51′–44° 05′ N, 127° 35′–127° 51′ E), is approximately 400 to 500 m above sea level and experience a temperate continental monsoon climate. The region has a mean annual temperature of approximately 3.5 °C and average annual precipitation of 700 to 800 mm. The soil type is dark brown soil with high fertility, and the forest type is mixed broadleaf-conifer that is composed primarily of coniferous trees such as *Pinus koraiensis* Sieb. et Zucc and *Abies holophylla* Maxim. and broadleaf trees such as *Fraxinus mandshurica* Rupr., *Juglans mandshurica* Maxim., *Acer mandshurica* Maxim., *Carpinus cordata*, *Tilia mandschurica* Rupr. et Maxim., and *Quercus mongolica* Fisch^47^.

Plot D10, located within the Xiaolong Mountains in Gansu Province (33° 30′–34° 49′ N, 104° 22′–106° 43′ E), belongs to a warm temperate and north subtropical transitional region that is approximately 1000 m above sea level. The region has a mean annual temperature of 7 °C to 12 °C and average annual precipitation of 460 to 800 mm. The soil type is humid dark brown mountain soil with a high organic content; the forest type is pine and oak mixed forest, containing primarily broadleaf trees such as *Quercus aliena* var. *acuteserrata* Maxim., *Quercus liaotungensis* Koidz., *Populus davidiana* Dode., *Toxicodendron vernicifluum* F.A. Berkley, *Populus purdomii* Rehd., *Tilia paucicostata* Maxim., *Carpinus cordata* Bl., *Crataegus kansuensis* Wils., and *Kalopanax septemlobus* Koidz. and coniferous trees such as *Pinus armandi* Franch. and *Pinus tabulaeformis* Carr.^47^.

Plot E11, located within the Jianfengling Nature Reserve in Hainan Province (18° 23′–18° 52′ N, 108° 46′–109° 02′ E), is 800 m above sea level and belongs to a tropical monsoon climate. The region has a mean annual temperature of approximately 23 °C and average annual precipitation of approximately 1150 mm. The soil type is lateritic yellow soil; the forest type is tropical montane rainforest, and the species diversity is high. The atypical dominant populations include *Cryptocarya chinensis*, *Gironniera subaequalis*, *Mallotus hookeriana*, and *Nephelium lappaceum*^47^.

### Uniform angle index (W)

**Figure 2:**
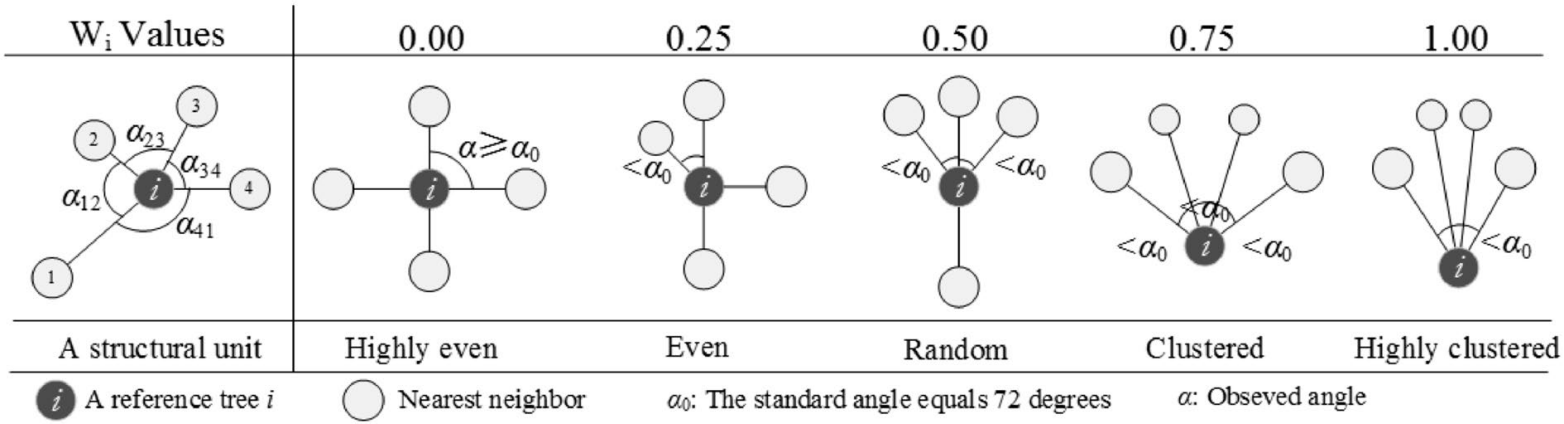
Principle of the structural unit (left) and the uniform angle index with k = 4 nearest neighbours and k + 1 = 5 possible discrete outcomes.

This study used the uniform angle index as the spatial structure parameter to analyse the distribution of neighbours of individuals^40,41^. The uniform angle index can be used to describe the uniformity of the nearest neighbours of a reference tree (Fig. 2, left). Two adjacent neighbours and a reference tree (i) form an angle (α_ij_). We measured the angles and compared the values with a standard angle ${\upalpha }_{0}$ (${\upalpha }_{0} (360^{ \circ } )/(n + 1)$, α_0_ = 72° with n = four neighbours) to analyse the distribution around the reference trees. W_i_ is defined as the proportion of smaller α_ij_ in the total number of angles^41^. The formula for this calculation is as follows:

$$\it {\text{W}}_{{\text{i}}} = \frac{1}{{\text{n}}}\sum\limits_{{{\text{j = 1}}}}^{{\text{n}}} {{\text{z}}_{{{\text{ij}}}} } {\text{,}}\,\,\,\,{\text{where z}}_{{{\text{ij}}}} = \left\{ {\begin{array}{*{20}l} {1,} \hfill & {\quad {\text{if}}\,{\text{the}}\,\alpha _{{{\text{ij}}}} \,{\text{angle}}\,{\text{is}}\,{\text{smaller}}\,{\text{than}}\,\alpha_{0} } \hfill \\ {0,} \hfill & {\quad {\text{otherwise}}} \hfill \\ \end{array} } \right.\,\,\,{\text{and}}\,\,\,\,{\text{ }}0 \leq W_{i} \leq 1$$

Different W_i_ values indicate different patterns for the nearest four neighbours around a reference tree^40^. Figure 2 shows the details of all W_i_ values and their appropriate meanings.

Each W_i_ value matches a different pattern of neighbours^40^. For convenience, we named the small pattern formed by the reference tree and its nearest four neighbours the *structural unit*^50^. Each structural unit is composed of five trees: one reference tree and four nearest neighbours. When W_i_ = 0 or 0.25, the structural units were categorized as even structural units, with the reference trees as the even trees; when W_i_ = 0.75 or 1, the structural units were categorized as clustered structural units, with the reference trees as the clustered trees; and when W_i_ = 0.5, the structural units were categorized as random structural units, with the reference trees as the random trees. Each tree was a reference tree and was included except trees in the buffer. The frequency of individuals and proportions of basal area based on different structural sub-types were calculated.

**Figure 3:**
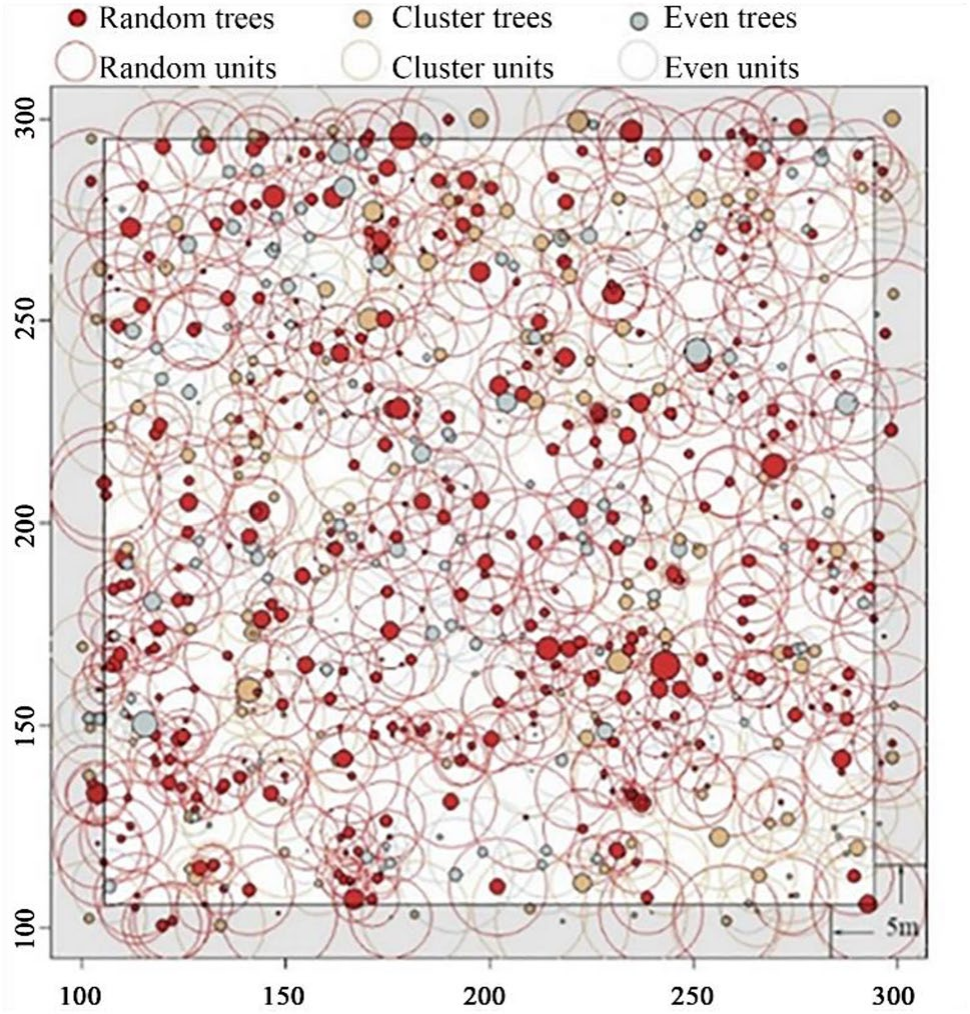
The structural units and buffer in Plot C4 as an example: shadow are the buffer, 5 m from each edge. Solid circles denote live trees, and their sizes are proportional to the DBH. X and Y are two perpendicular coordinate axes of the plot. All DBH is enlarged 2.5 times for viewing purposes^47^. This figure was generated by R (version 3.5.1, R Development Core Team 2020).

An example of different types of trees and structural units is shown in Fig. 3.

### Sub-types within the random structural units

**Figure 4:**
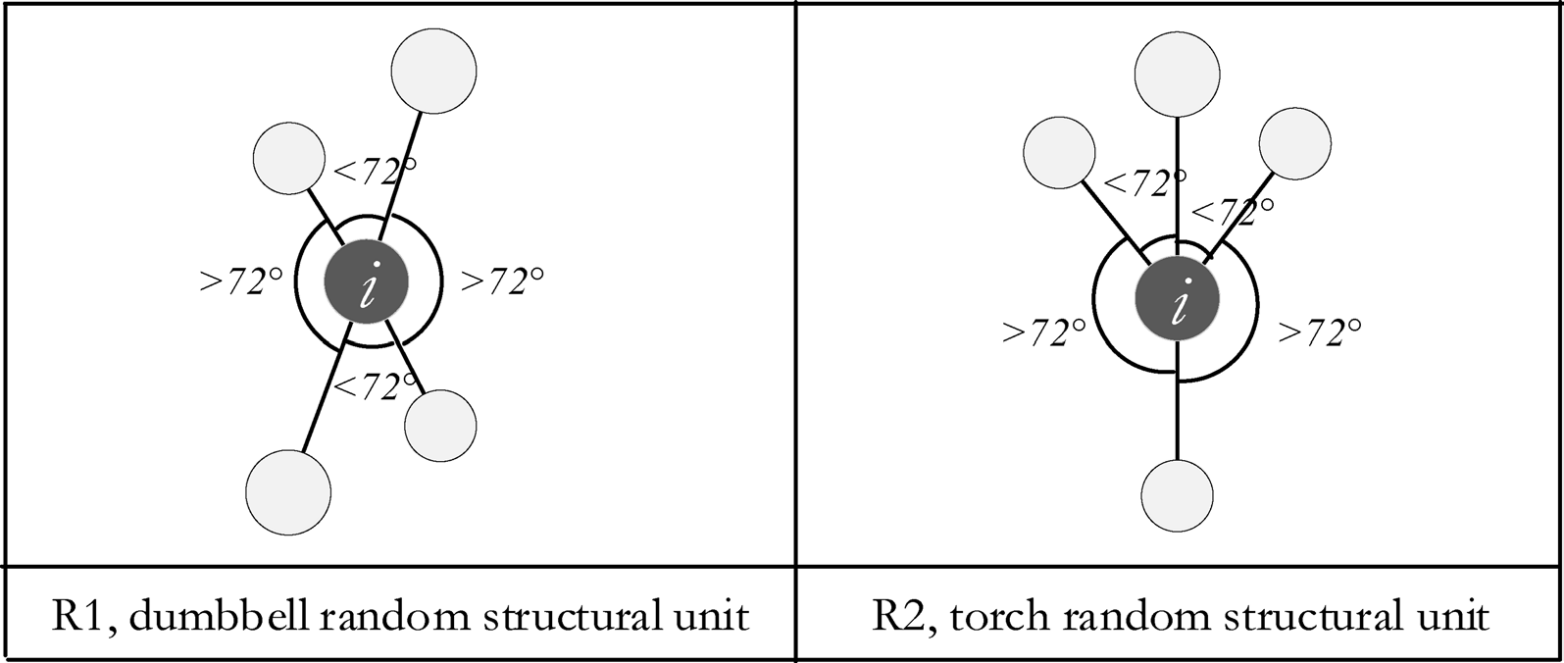
Different types of random structural units according to the uniform angel index.

The central W_i_ value of random structural units is 0.5 according to the definition based on the uniform angle index. Starting from the reference tree, four adjacent trees and the reference tree form four angles (α_i_: α_12,_ α_23,_ α_34,_ and α_41_), as shown in Fig. 2. In the structural units of random type, two angles are less than the standard angle α_0_ (α_0_: 72 degrees), while the other two are greater. The two possible distributions of these four angles are as follows:

**Type R1:** In any two adjacent angles, one is less than α_0_, while the other is greater or equal, that is, Z_ij_ = 0 while Z_j(j + 1)_ = 1, or Z_ij_ = 1 while Z_j(j + 1)_ = 0. In formula (1), ${\sum }_{j=1}^{4}{Z}_{ij}=\left(1+0+1+0\right)$ or ${\sum }_{j=1}^{4}{Z}_{ij}=\left(0+1+0+1\right)$. We call this type of random structural unit type R1, and the corresponding reference trees are R1 random trees, as shown in Fig. 4, left. We also call this type a “dumbbell” unit, as the shape is similar to a dumbbell.

**Type R2:** Two adjacent angles can be found that are less than α_0,_ while the other two are greater or equal. Therefore, Z_ij_ = 0 while Z_j(j+1)_ = 0, or Z_ij_ = 1 while Z_j(j+1)_ = 1. Formula (1) can be presented as ${\sum }_{j=1}^{4}{Z}_{ij}=\left(1+1+0+0\right)$, ${\sum }_{j=1}^{4}{Z}_{ij}=\left(0+0+1+1\right)$, ${\sum }_{j=1}^{4}{Z}_{ij}=\left(1+0+0+1\right)$ or ${\sum }_{j=1}^{4}{Z}_{ij}=(0+1+1+0)$. We call this type of random structural unit type R2, the corresponding reference trees are R2 random trees, as shown in Fig. 4, right. We also call this type a “torch” unit, as the shape is similar to a torch.

To avoid systematic errors from trees near the stand edge, we set a 5 m buffer for each plot (Fig. 3). Trees near the edges were calculated as neighbours only but not a reference tree. R (version 3.3.1 https://www.r-project.org/) was used to calculate the basal area of individuals and structural units^47^.

The beanplot was used to show the results. Plots beans to compare the distributions of different groups; it draws one bean per group of proportions. A bean consists of a one-dimensional scatter plot, its distribution as a density shape and an average line for the distribution. Next to that, an overall average for the whole plot is drawn per default (https://www.rdocumentation.org/packages/beanplot/versions/1.2/topics/beanplot).

## Results

### Proportions of the frequency of the sub-types

**Figure 5:**
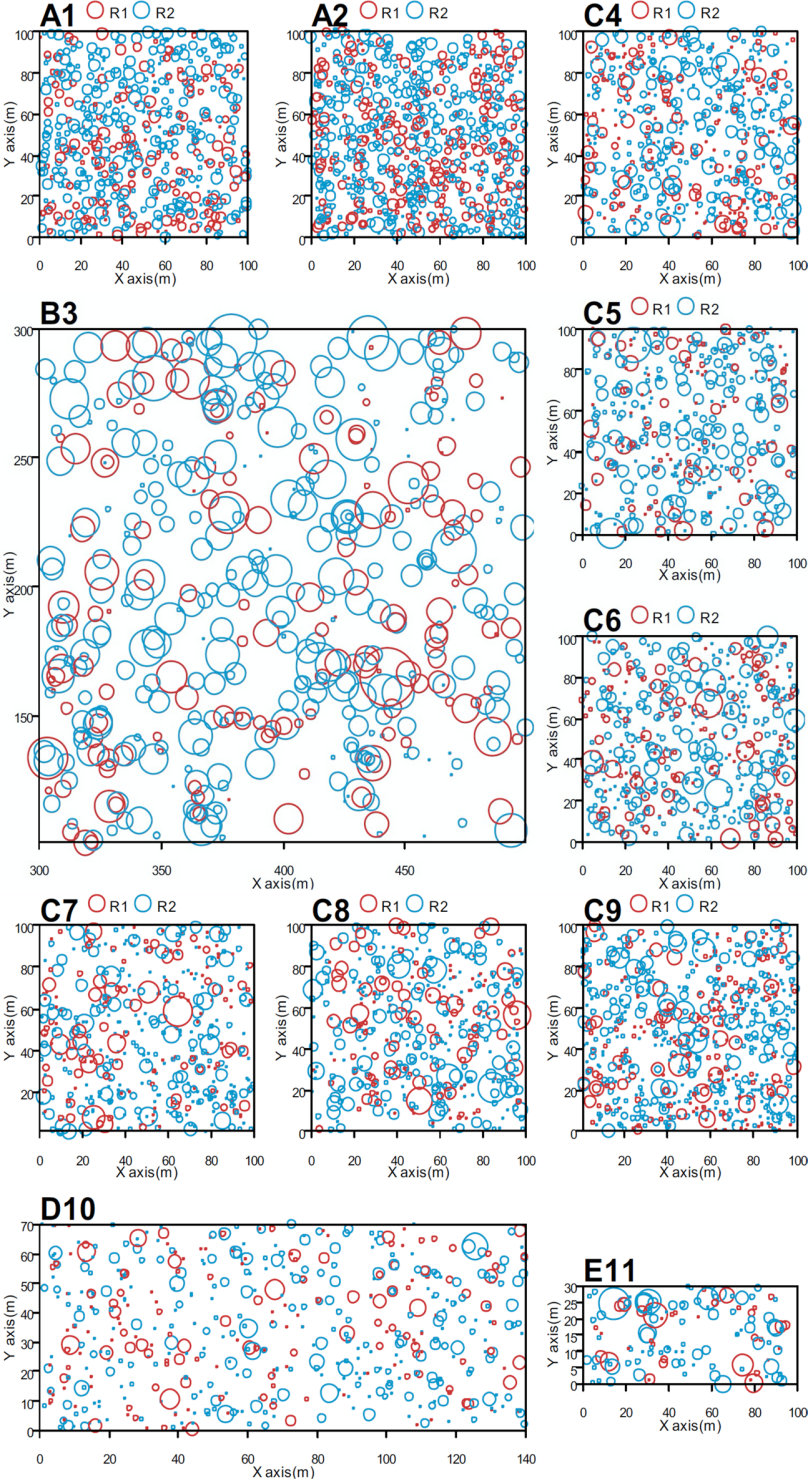
Plot maps with trees classified according to the sub-type structural unit. Clustered and even structural units are not shown in the maps.

Considering four neighbours in the evaluation of the uniform angle index^41^, there are only two types of random structural units in the horizontal distribution: R1 and R2. In all natural forest plots, we found both sub-types simultaneously exist (Fig. 5). The frequencies of R1 units in the sample plots were generally less than those of R2. The average proportion of R1 in the 11 plots was 33.25% (standard deviation = 2.28), approximately 1/3 of the total; the average for R2 was greater, ranging from 61.92% to 70.11%, the average value of the 11 plots was 66.75%, approximately 2/3.

### Proportions of basal area of the sub-types

**Figure 6:**
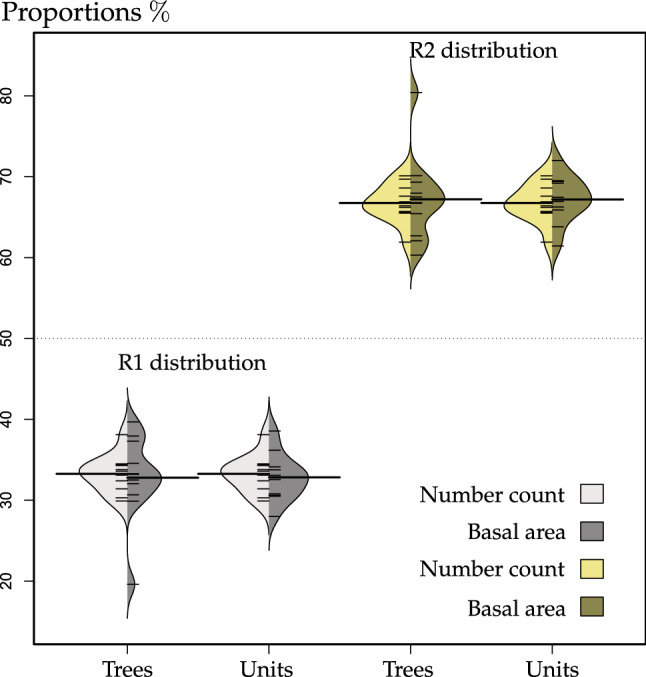
Proportions and distributions of R1 and R2 random trees and units based on the number of individuals or basal area.

A beanplot presents the proportions and distributions in all 11 plots, see Fig. 6. The basal area of the reference trees was calculated and the structural units including five trees as well.

R2 had the opposite distributions of that R1 since the total proportion equalled 1 in each stand. The proportions based on numbers were the same when calculating the individuals and basal area. This was because the structural units were defined according to the reference trees^51^.

The average proportions of R1 based on basal area was 32.80% (reference trees) and 32.82% (structural units) respectively. We found the lowest proportion of R1 was 19.68% appeared in plot C5, showing as a “tail” in Fig. 6. The distribution of reference trees based on basal area had the largest standard deviation (5.34). However, it fell to 2.91 when the result turned to the structural units. The “tail” was missing, the proportion of R1 increased to 28.00% in plot C5.

Consequently, three distributions of the proportions did not significantly differ according to the ANOVA test (Table 2).

**Table 2:** P values of ANOVA test among three distributions.

	P1	P2	P3
P1	–	0.793	0.701
P2	–	–	0.986

Proportions of R1 in 11 plots: P1, proportions of reference trees/structural units based on the frequency of numbers; P2, proportions of reference trees based on basal area; P3, proportions of structural units based on basal area.

## Discussion

Many processes such as biotic or interactions between biotic and abiotic processes operate simultaneously and may result in the complex spatial patterns that lead to high structural diversity^52,53^, which depends on spatial relations among neighbours. This study used the nearest neighbour summary statistics (NNSS) to describe relationships between a point and the *k* nearest neighbours^54^. There are three different possibilities of expressing location diversity with NNSS including distances (e.g. the aggregation index R'^55^), angles (e.g. the means of angles index^56^) and directions (e.g. the mean directional index^57^). One of the advantages of angle-based approaches is the distance between individuals is not necessary, which means stand density does not affect the pattern. The uniform angle index is one of NNSS using angles as location test. Recently, the uniform angle index with four nearest neighbours has been developed as a classic method, which has been widely used for both point pattern analysis and practical applications of forest management^43,47,58^.

Three to ten or more nearest neighbours could be chosen according to the definition of the uniform angle index^59^. Some studies discussed the effect of different numbers of neighbours. Wang et al. proposed that the four-tree structural unit is the best compromise between sampling accuracy and costs for practical forest management or testing the complete spatial randomness hypothesis^60^.

### Proportion of structural types in natural forests

There are currently many forest classification methods, such as the Kraft classification method^61^, IUFRO classification method^62^, Hawley tree classification method^63^, etc., these methods all focus on the characteristics of trees instead of the distribution of the neighbours of individuals. The study of Zhang et al.^47^ shed new light on the application of the uniform angle index, which can be used not only for the determination of horizontal patterns but also, more importantly, for the division of different structural types according to the distribution of neighbours in forest communities.

**Figure 7:**
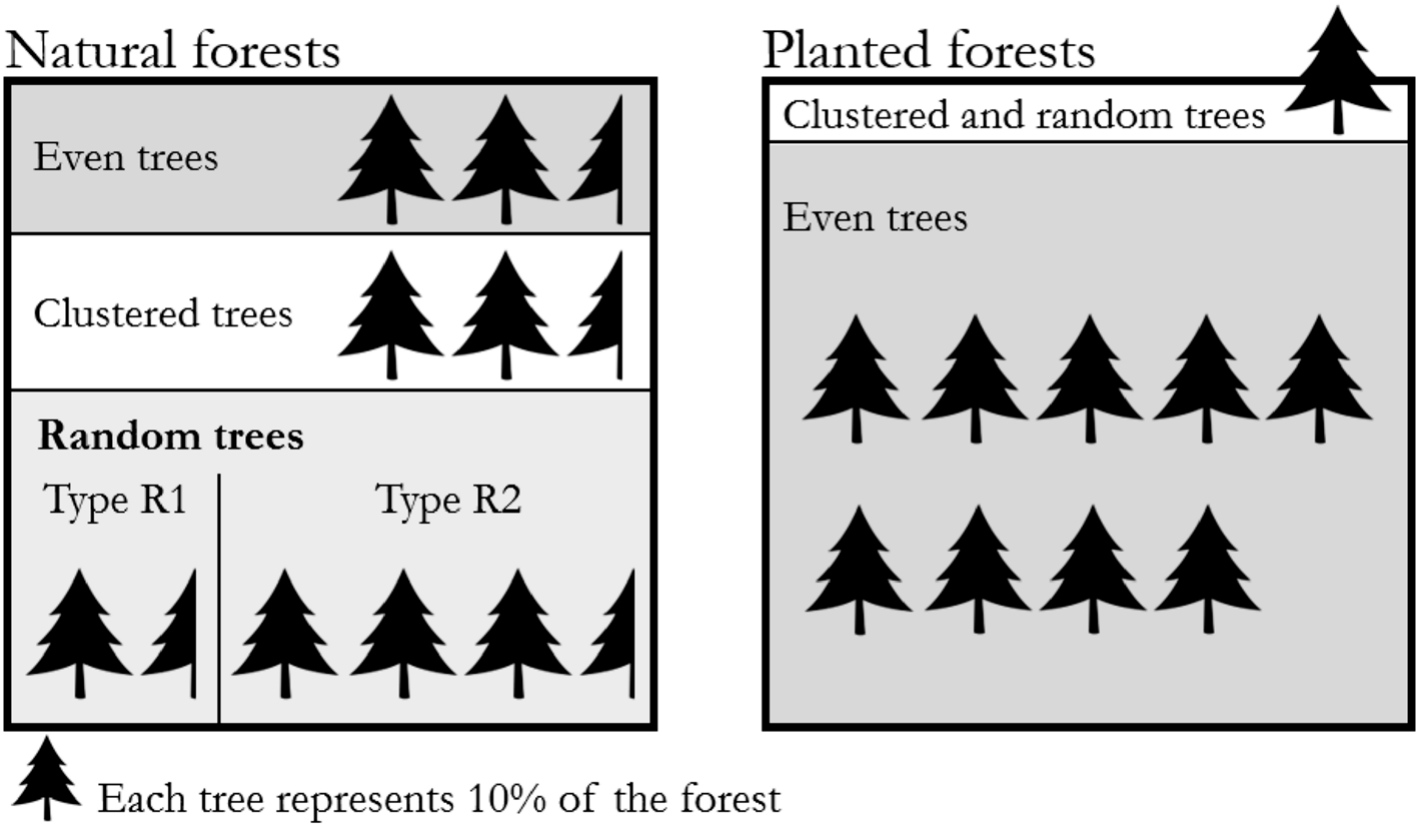
The proportion of structural types in natural and planted forests. The number of “trees” shows the approximate proportion of different types.

This study determined the different types in natural forests and attempted to provide answers in combination with previous studies. Imagine a forest is a “box”. What is the proportion when considering the structural types in a natural forest? What is the difference compared with planted forests when only considering the distribution of neighbours of individuals? Fig. 7 offers a graphical comparison of the characteristic distributions of structural units in natural and planted forests. Natural forests showed a more complex composition regarding structure diversity. According to Zhang et al.^47^, more than 50% of trees are randomly distributed. In this study, we also determined that both the frequency and basal area of the R1 trees were less than those of the R2 trees, accounting for approximately one-third of the total number of random units in the forests. Those of the R2 type were approximately twice those of the R1, accounting for approximately two-thirds of all random units. Regardless of the reference trees or the complete structural units as the statistical object, the final result showed that the random structural units were mainly composed of type R2 (torch type).

### Clarification on the near-natural cultivation of planted forests

The productivity and stability of plantations have long been considered key issues. The spatial structure and resulting structural differences of forests determine the habitat and species diversity of forests and determine the stability of forest ecosystems^48^. In Europe, there are several calls for nature-based forest management in both natural forest and plantations^64^. In Australia, the trend is towards the phasing-out of native forest harvesting in favour of plantations^65^, preferably with mixtures of indigenous species to increase complexity^66^. Many processes such as disturbance, regeneration, and differential thinning may result in complex spatial patterns. For example, in the Mediterranean region, mixed conifer-broadleaf natural forests were damaged by overgrazing, over-harvesting and fire. The artificial forests were established at the end of the nineteenth century to rehabilitate this area. However, after thinning and harvesting of the planted forests, forests reverted naturally to that are similar in structure and species composition to the natural forests that existed before their degradation. Undoubtedly, it will take a long time for forests returning to the natural states.

In analysing the different types of natural forests in this paper, we found that the diversity differences between plantations and natural forests were not only species composition but also in the aspect of spatial structure^67^. Unlike natural forests, shown in Fig. 7, planted forests are much simpler, with even trees as the major type. Random and clustered trees occurred rarely^47^. A simple spatial structure is now a major challenge facing plantations in various countries.

The purpose of our series of studies was to take the spatial structure of natural forests as a template and apply the structural diversity of natural forests to plantations to decrease the gap between planted forests and natural forests. After the detailed classification of random trees has been determined, these details will be expected to be added to the management of plantations, and the method will be imitated to guide near-natural planted forests or to adjust large-diameter trees in existing forests to a more complex structure. Increasing the diversity of plantations by human intervention will effectively change the single spatial structure of plantations and improve their understory micro-environment to improve productivity and stability. Here are two suggestions for different plantations: (1) Random structural units could be included in a newly planted forest instead of planting all regular structural units. (2) Existing plantations are recommended to form random structural units by thinning activities during the forest management, but without replantation.

## Conclusion

This study involved 11 natural forest plots that were distributed in different latitudes or regions of China. The forest types were different, including mixed and pure forests. The forest patterns varied, including even, random and clustered distributions^40^. The proportions of random trees revealed by the uniform angle index were related neither to the regional distribution and forest types nor to the composition of tree species and forest spatial pattern. To further explore the specific structural types of random structural units, the trees were divided into two types according to their characteristics using the uniform angle index.

In^38^ 72 degrees has been determined as the angular threshold "producing an average value of W = 0.5 for a random distribution"^56^ hence, in structural units of random type there are two pair of neighbours forming angles wider than the threshold and two forming smaller angles. In R1 sub-type the distribution of the pairs is alternated (larger, smaller, larger, smaller) while in R2 sub-type the pairs are adjacent (larger, larger, smaller, smaller). The analysis showed that both kinds of random units exist naturally in forests. Therefore, the spatial formation mechanism of forest communities and structural diversity is interpreted using our simple approach, which is of great significance and meaning to the cultivation of artificial forests.

## Supplementary information

Supplementary Information 1.
