# Supplementary material for: A simple and effective approach to quantitatively characterize structural complexity
Source: Sci Rep. 2021 Jan 14;11:1326. doi: 10.1038/s41598-020-79334-7 (PMC7809123; doi:10.1038/s41598-020-79334-7)
Supplement: Supplementary file 1 — Supplementary Information 1. [file 41598_2020_79334_MOESM1_ESM.docx]

Appendix:

Title page

A simple and effective approach to quantitatively characterize structural complexity

**Gongqiao Zhang^1^, Gangying Hui^1*^, Aiming Yang^1^, Zhonghua Zhao^1^**

Research Institute of Forestry, Chinese Academy of Forestry, Key Laboratory of Tree Breeding and Cultivation of National Forestry and Grassland Administration, Box 1958, Beijing 100091, China

*Corresponding author: Gangying Hui; Email: [hui@caf.ac.cn](mailto:hui@caf.ac.cn)

**Information on the Study Area**

| **Plot** | **Location** | **Elevation**  **/m** | **Climate** | **mean annual temperature**  **/centigrade** | **Average Annual precipitation**  **/mm** | **Mixed/Pure** | **Soil type** |
| --- | --- | --- | --- | --- | --- | --- | --- |
| A1, A2 | 47°36’ – 48°35’N, 118°58’ – 120°32’E | 700 - 1100 | Mid-temperate, semi-humid, and semiarid continental monsoon climate | 1.5 | 344 | Pure *Pinus sylvestris* var. *mongolica* forests | Sand |
| B3 | 43°59’ – 43°28’N, 87°12’ – 87°50E’ | 1635 - 1706 | Temperate continental climate | 5 - 7 | 600 - 800 | *Picea schrenkiana* natural forest with very few *Betula tianschanica* | Mountain grey cinnamon forest soil |
| C4 -C9 | 43°51’ – 44°05’N, 127°35’ – 127°51’E | 400 - 500 | Temperate continental monsoon climate | 3.5 | 700 - 800 | Pine and oak mixed forest | Dark brown soil |
| D10 | 33°30’ – 34°49’N, 104°22’ – 106°43’E | 1000 | Warm temperate and north subtropical transitional climate | 7 - 12 | 460 - 800 | Pine and oak mixed forest | Humid dark brown mountain soil |
| E11 | 18°23’ – 18°52’N, 108°46’ – 109°02’E | 800 | Tropical monsoon climate | 23 | 1150 | Tropical montane rainforest with high species diversity | Yellow soil |
